# Efficacy of an imidacloprid/flumethrin collar against fleas, ticks and tick-borne pathogens in dogs

**DOI:** 10.1186/1756-3305-6-245

**Published:** 2013-08-23

**Authors:** Filipe Dantas-Torres, Gioia Capelli, Alessio Giannelli, Rafael Antonio Nascimento Ramos, Riccardo Paolo Lia, Cinzia Cantacessi, Donato de Caprariis, Anna Sara De Tommasi, Maria Stefania Latrofa, Vita Lacasella, Viviana Domenica Tarallo, Giancarlo Di Paola, Barbara Qurollo, Edward Breitschwerdt, Dorothee Stanneck, Domenico Otranto

**Affiliations:** 1 Department of Veterinary Medicine, University of Bari, Bari, Valenzano, Italy; 2 Current address: Department of Immunology, Aggeu Magalhães Research Center, Recife, Pernambuco, Brazil; 3 Istituto Zooprofilattico Sperimentale delle Venezie, Padova, Legnaro, Italy; 4 Center for Biodiscovery and Molecular Development of Therapeutics, James Cook University, Cairns, Australia; 5 Intracellular Pathogens Research Laboratory, Center for Comparative Medicine and Translational Research, College of Veterinary Medicine, North Carolina State University, Raleigh, NC, USA; 6 Bayer Animal Health GmbH, Leverkusen, Germany

**Keywords:** Canine vector-borne diseases, *Anaplasma platys*, *Babesia vogeli*, *Hepatozoon canis*, Prevention, Dog, Imidacloprid, Flumethrin, Tick

## Abstract

**Background:**

Tick-borne diseases comprise a group of maladies that are of substantial medical and veterinary significance. A range of tick-borne pathogens, including diverse species of bacteria and protozoa, can infect both dogs and humans. Hence, the control of tick infestations is pivotal to decrease or prevent tick-borne pathogen transmission. Therefore, different commercial products with insecticidal, repellent or both properties have been developed for use on dogs. Recently, a collar containing a combination of imidacloprid 10% and flumethrin 4.5% has proven effective to prevent tick and flea infestations in dogs under field conditions and the infection by some vector-borne pathogens they transmit under laboratory-controlled conditions.

**Methods:**

From March 2011 to April 2012, a field study was conducted in a private shelter in southern Italy to assess the efficacy of the imidacloprid/flumethrin collar against tick and flea infestations and to determine if this strategy would decrease tick-borne pathogen transmission in young dogs. A total of 122 animals were enrolled in the study and randomly assigned to group A (*n* = 64; collared) or group B (*n* = 58; untreated controls). Dogs were examined monthly for ticks and fleas and systematically tested for selected tick-borne pathogens.

**Results:**

Compared to controls, the collar provided overall efficacies of 99.7% and 100% against tick and flea infestation, respectively. The overall efficacy for the prevention of tick-borne pathogens (i.e., *Anaplasma platys* and *Babesia vogeli*) was 91.6%.

**Conclusions:**

This study demonstrates that the imidacloprid/flumethrin collar is efficacious against flea and tick infestation as well as tick-borne pathogen transmission to dogs under field conditions.

## Background

Tick-borne diseases (TBDs) comprise a group of illnesses caused by numerous pathogens (e.g., viruses, bacteria, protozoa, and helminths) that may be transmitted by a number of tick species [1]. These diseases may range from subclinical to life-threatening conditions, with the severity of clinical signs depending on the microorganism(s) involved and the host immune response against the infection [2,3]. Importantly, some pathogens transmitted by certain tick species belonging to the *Rhipicephalus sanguineus* group are primarily found in association with dogs, but may potentially infect humans, as is the case of *Ehrlichia canis*, *Rickettsia conorii*, *Rickettsia massiliae*, and *Rickettsia rickettsii*[1]. These observations highlight the importance of controlling tick infestations in dogs, so as to reduce the risk of tick-borne infections in pets and family members.

The Mediterranean region provides a suitable environment for the development of a range of tick species, which infest dogs throughout the year. Consequently, some TBD-causing pathogens (e.g*., Anaplasma platys*, *Babesia vogeli*, *E. canis* and *Hepatozoon canis*) are widespread and highly prevalent in some Mediterranean countries [2,4-6]. In highly endemic areas, the typical clinical presentation associated with individual TBDs in dogs may potentially be masked by simultaneous infections by multiple pathogens [3,7].

Clearly, the risk of contracting TBDs is directly associated with the exposure to tick vectors. Therefore, the use of insecticides and repellents is currently considered the best option to prevent infections by tick-borne pathogens in dogs [8]. For instance, a spot-on formulation containing 10% imidacloprid and 50% permethrin (Advantix®, Bayer HealthCare AG, Germany) has proven effective in protecting dogs against tick infestations under field conditions, as well as in preventing the transmission of selected tick-borne pathogens [9,10]. Recently, a collar containing 10% imidacloprid and 4.5% flumethrin (Seresto®, Bayer Animal Health, Germany) was developed for use on dogs and cats. This product contains both repellent and insecticidal properties and has proven effective against fleas, ticks, mites and lice [11-15]. The imidacloprid/flumethrin collar was highly efficacious in curing animals living in communities highly infested with ticks and fleas and to prevent reinfestations for up to 8 months in a refuge with a history of unsuccessful environmental tick control [16].

Furthermore, recent laboratory studies have demonstrated the efficacy of this collar for the prevention of vector-borne pathogen transmission in both dogs and cats [17-20]. Specifically in dogs, this collar was effective in preventing the transmission of *Babesia canis* and *E. canis* by ticks [18,19] as well as of *Dipylidium caninum* by the cat flea *Ctenocephalides felis*[17], under laboratory-controlled conditions. More recently, this collar was proven highly efficacious against *L. infantum* infection under field conditions [21]. Herein, we assessed the efficacy of this new device against tick and flea infestation as well as its efficacy against selected TBD pathogens (i.e., *A. platys*, *B. vogeli*, and *H. canis*) in young sheltered dogs living in an area where these infections are highly prevalent.

## Methods

### Study design and study area

A parallel group-designed, randomised, controlled efficacy field trial was conducted in a private shelter in the municipality of Putignano (40°51′ N, 17°7′ E, 372 m above sea level), province of Bari, Apulia region (southern Italy). The ectoparasite fauna and TBDs occurring in dogs in this shelter had been monitored over the previous three years [1,10,22].

The design and experimental procedures used in this study were authorized by the Italian Ministry of Health (DGSA n° 0001997; 04/02/2011). Moreover, this study was conducted in accordance with the principles of Good Clinical Practice (VICH GL9 GCP, 2000) adopted by the Committee for Medicinal Products for Veterinary Use (CVMP) in the guidelines for the evaluation of the efficacy of antiparasitic substances against tick and flea infestation in dogs and cats (EMEA/CVMP/005/00, 2000).

### Animal management and care

Dogs enrolled in this study were young dogs (≤6 months old) of both sexes. Each dog received two doses of a vaccine against canine parvovirus, adenovirus type 2, distemper virus, *Leptospira canicola* and *Leptospira icterohaemorrhagiae* (Duramune® DAPPi + LC; Zoetis, Italy). In addition, all dogs were dewormed with a combination of praziquantel, pyrantel pamoate and fenbantel (Drontal plus®; Bayer AG, Germany) at enrolment, and were examined every two months and treated whenever a faecal sample from a given cage was found positive for intestinal parasite eggs.

Dogs were housed in wire mesh enclosures (approx. 10 × 20 m) and fed commercial feed once per day, with water provided *ad libitum*. At each treatment time point (see below), the clinical status of each dog was recorded on individual forms. The application of other ectoparasiticides was not allowed during the study period except once (i.e., June 2011), when heavy tick infestation was recorded in untreated dogs.

### Enrolment and follow up assessments

Dogs (*n* = 176) were enrolled into the study between March and May 2011. On day 0 each dog was microchipped, photographed, clinically examined and searched for ticks and fleas. All data, including sex, age, weight, and coat length, were recorded in appropriate individual files. Dogs were excluded from the study if under 7 weeks of age, if skin lesions were observed at the site of product application or if physical examination revealed a pre-existing disease.

The 176 enrolled dogs were randomly assigned to groups A (dogs collared at day 0) and B (untreated control dogs), but 52 of them died during the first weeks following enrolment due to parvoviral gastroenteritis (data not shown). The remaining dogs, reaching the first follow-up assessment (see below), consisted of 64 dogs in group A and 58 in group B. The homogeneity of the two groups in relation to sex, age, weight, and coat length was evaluated using chi-square test and one-way ANOVA.

Blood and serum samples were collected from all dogs included in the trial at the first (July 2011), second (September 2011), third (November 2011) and fourth (April 2012) follow-up assessments.

All dogs were enrolled in the trial regardless of the results of the initial testing for the selected pathogens (see below). The inclusion of positive dogs in both groups was aimed at facilitating the circulation of pathogens within the dog population under examination, as well as at assessing the efficacy of the ectoparasiticide treatment at the subsequent follow-ups. At baseline (March-May 2011) collars were applied to all dogs from group A, according to the manufacturer’s instructions: dogs ≤8 kg received a small collar and dogs >8 kg received a large collar.

The collars were constantly worn and were replaced within two days in case of accidental losses. In addition, a new (large) collar was applied to dogs whose body weight increased to over 8 kg. Finally, all collars were replaced 8 months after application, as per manufacturer’s instructions. All occurrences (collar replacement, etc.) were recorded on the dog’s individual file. At the end of the study period (April 2012), all collars were removed from group A dogs and in October 2012 a complete blood sample collection was performed to determine the incidence of TBDs amongst dogs previously collared (group A) or not collared (group B). The laboratory staff were blinded to treatment allocation of individual dogs, whereas the shelter staff were not blinded due to the visible collar.

### Estimation of flea and tick load

Dogs were examined monthly for the presence of ticks and fleas by thumb counting, with examination of the following body regions: head, ears, breast-neck, thorax, abdomen, fore and back limbs, inter-digital areas, axilla, tail and inguinal area. The number and/or developmental stages of ticks and fleas detected in the above mentioned body sites were recorded on a separate form for each dog. Adult ticks were counted, whereas, due to the exceedingly large number of immature ticks, a visual estimate was made of the ticks located in the neck, thorax, abdomen, inter-digital and periocular areas and ears. Each site was considered as an independent unit of calculation. The load of immature ticks was grouped into the following four infestation classes:

• Low: <10 immature ticks;

• Medium: <10x < 50 immature ticks;

• High: <50x < 100 immature ticks;

• Very high: ≥100 immature ticks.

Each of the four infestation classes was then multiplied by the number of body sites in which that parasitic load was estimated. Representative tick and flea specimens were identified based on morphology [23-25].

### Sample collection and diagnostic tests

Blood samples were collected from the brachial or jugular veins in tubes with and without ethylenediaminetetraacetic acid (EDTA) anticoagulant. Room temperature coagulated blood was centrifuged at 1,678 *g* for 10 min prior to collecting the separated serum. Both the EDTA whole blood and serum samples were stored at –20°C until tested (see below).

Antibodies against *B*. *canis* and *E*. *FLUOBABESIA canis* were detected using commercial IFAT kits (MegaScreen Fluobabesia, MegaCor GmbH, Austria, and Canine Ehrlichiosis FA Substrate Slide, VMRD, Pullmann, Washington, USA, respectively). Cytological examination of whole blood and buffy coat smears was performed, following staining with MGG Quick Stain (Bio Optica, Milan, Italy), for the presence of intracellular inclusions of the most common tick-borne pathogens of dogs.

For the detection of *Ehrlichia* and *Anaplasma* species, total DNA was extracted from EDTA-blood samples using MagAttract DNA Blood Mini M48 Kits and BioRobot M48 Workstation (Qiagen, Valencia, CA, USA). DNA samples were screened by amplification of a fragment of the *groEL* gene and a fragment of the 16S rRNA gene, which are conserved across all *Ehrlichia* and *Anaplasma* species [26,27]. All positive samples were either sequenced directly (Genewiz, Inc., Research Triangle Park, NC) for speciation or subsequently tested by PCR assays designed for the detection of specific genetic markers for *A. platys*[28], *A. phagocytophilum*[29], and *E. canis*[10]. *Babesia* spp. DNA was amplified by PCR targeting a variable region of the 16S-like rRNA gene [30] and *H. canis* DNA by a PCR targeting a fragment of the 18S rRNA gene [31], as described previously. Finally, *Bartonella* spp. was amplified by PCR targeting a fragment of the RNA polymerase b subunit (*rpoB* gene) as described elsewhere [32].

### Statistical analysis

The minimum sample size (*n* = 49) was calculated using the software WinEpi (http://www.winepi.net/uk/index.htm), to estimate differences between proportions (i.e., incidence) from the two populations. The following assumptions were considered: (I) expected incidence in group A = 5%; (II) expected incidence in group B = 15%; (III) power = 85%; (IV) level of confidence = 95%. To accommodate potential losses during the study period, ~60 instead of 49 dogs were enrolled in each group.

The incidence of tick-borne pathogen infection was determined by Incidence Density rates (IDRs) [10,33], which was calculated as follows: IDRs = number of positive dogs/number of dog-months of follow-up (i.e., the number of months between the previous and the following assessment for each dog at risk of infection). Dogs were defined as “positive” if positive at any of the cytological, serological or PCR tests performed. Differences between IDRs in groups A and B were calculated using a Yates-corrected chi squared test. Dogs tested only once (e.g., those that died from parvoviral gastroenteritis) did not contribute to the calculation of incidence, whereas those sampled at least twice contributed to the IDR calculation during the number of months those dogs remained in the study. The post-treatment incidence was calculated considering the test results obtained in October 2012 in dogs that were negative both at enrolment and April 2012 prior to the removal of the collar.

The efficacy of the collar against tick-borne pathogens was calculated as follows: Efficacy = [(% of positive dogs in group B − % of positive dogs in group A)/% of positive dogs in group B] × 100. For the calculation of the overall efficacy against tick-borne pathogens, *H. canis* was excluded, as this protozoon is not transmitted by tick bites. The efficacy of the collar against ticks (immature and adult stages) and fleas was calculated as follows: Efficacy = (mean ectoparasites load on control dogs – mean ectoparasites load on treated dogs)/(mean ectoparasites load in control dogs) × 100. Collars were deemed effective against ectoparasites if the calculated efficacy, based on arithmetic and geometric means, was at least 90% [34].

## Results

Dogs from group A (*n* = 64) and group B (*n* = 58) were homogeneous (p > 0.05) in terms of number and individual characteristics (i.e., sex, age, weight, and coat length). At baseline, 25 (20.5%) out of 122 dogs were positive for at least one tick-borne pathogen. Specifically, 17.2% of the dogs were infected with *A. platys* (i.e., 22% vs. 12% in groups A and B, respectively), 11.5% with *H. canis* (i.e., 10.9% vs. 9.1%), 4.9% with *Babesia* spp. (4.7% vs. 5.2%) and 1.6% with *Bartonella* spp. (3.1 vs. 0%). The pathogen prevalence at baseline between the two groups was not significantly different.

Two dogs were infected with *Bartonella vinsonii* subsp. *berkhoffii* genotype III and one dog each was infected with *Bartonella henselae* and *Bartonella rochalimae.* Co-infections by two or more pathogens were documented in 16 dogs (13.1%), with seven dogs infected by *A. platys* and *H. canis*, two dogs with *A. platys* and *B. vogeli* and two dogs with *A. platys* and *B. vinsonii* subsp. *berkhoffii* genotype III. In three dogs, co-infection by three pathogens was diagnosed (data not shown). All dogs were negative for *A. phagocytophilum* and *E. canis* and only two dogs were PCR-positive for *B. vinsonii* subsp. *berkhoffii* genotype III and *B. rochalimae* in group B. Thus, the efficacy of the collar against these pathogens could not be calculated.

The IDRs for all tick-borne pathogens at all follow-up assessments were significantly higher in group B than in group A (Table 1), resulting in an overall efficacy of 91.6%. For individual pathogens the efficacies were as follows: 100% for *B. vogeli*; 91.1% for *A. platys*; and 43.4% for *H. canis*. At the end of the trial (April 2012), 110 dogs (61 from group A and 49 from group B) remained untreated and the post-treatment incidence, calculated in October 2012 (Table 2), indicates *A*. *platys* as the most common pathogen (incidence of 67.4% and 31% in groups A and B, respectively), followed by *H. canis* (9.6% and 47.8%), *B. vogeli* (5.1% and 34.8%), and *B. vinsonii* subsp. *berkhoffii* genotype III (4.1% in group B only). Co-infections were detected in 25 (20.5%) dogs, with eight dogs infected by *A. platys* and *H. canis*, eight with *B*. *vogeli* and *H. canis*, six with *A*. *platys* and *B. vogeli*, and three with *A*. *platys*, *B. vogeli* and *H. canis*.

**Table 1:** **Incidence density rates (IDRs) of and efficacy (%) against *Anaplasma platys*, *Babesia vogeli *and *Hepatozoon canis *infections in dogs from groups A and B**

**Pathogen**	**Number of dogs in the cohort**		**Number of new infections**		**Dog-months of follow-up**		**IDRs**		**Efficacy**
	**A**	**B**	**A**	**B**	**A**	**B**	**A**	**B**	
*Anaplasma platys*									
Baseline	64	58	-	-	-	-			
Follow-up 1	50	51	6	24	134.5	109.7	53.5	262.6	
Follow-up 2	44	23	4	18	115.7	59.8	41.5	361.2	
Follow-up 3	40	5	0	2	82.8	12.5	0	192.8	
Follow-up 4	40	3	0	0	162.8	12.5	0	0	
Total			10	44	495.8	194.4	24.2	271.6	91.1%
*Babesia vogeli*									
Baseline	64	58	-	-	-	-	-	-	
Follow-up 1	64	55	0	18	177.3	121.0	0	178.5	
Follow-up 2	63	35	0	1	165.7	91.7	0	13.1	
Follow-up 3	63	33	0	2	129. 8	68.0	0	35.3	
Follow-up 4	63	30	0	0	256.4	120.3	0	0.0	
Total			0	21	729.2	401.0	0	62.9	100%
*Hepatozoon canis*									
Baseline	64	58	-	-	-	-	-	-	
Follow-up 1	57	51	25	27	156.8	109.7	191.4	295.5	
Follow-up 2	32	23	10	8	84.5	59.3	142.1	161.8	
Follow-up 3	22	15	1	4	45.5	31.1	26.4	154.6	
Follow-up 4	21	11	0	3	85.5	45.7	0.00	78.9	
Total			36	42	372.2	245.7	116.1	205.1	43.4%

**Table 2:** Incidence of canine vector-borne pathogen infections in the former groups A (treated) and B (untreated), six months after the end of the study (October 2012)*

**Pathogen**	**Positive/Total (%)**		**Total (%)**
	**Former group A**	**Former group B**	
*Anaplasma platys*	33/49 (67.3)^*^	10/30 (33.3)^*^	43/79 (54.4)
*Babesia vogeli*	3/59 (5.1)	5/34 (14.7)	8/93 (8.6)
*Bartonella* spp.	0/60 (0.0)	2/48 (4.2)	2/109 (1.8)
*Hepatozoon canis*	5/52 (9.6)^*^	11/23 (47.8)^*^	16/75 (21.3)

*Statistically significant differences (p < 0.01) between former groups A and B.

The mean tick and flea load in both group A and B were comparable at baseline (p > 0.05). Conversely, the tick burden was significantly higher in control dogs (p < 0.01) throughout the study period, resulting in an overall efficacy against tick attachment of 99.7%, ranging from 95.3 to 100% and from 98.2 to 100% against adult and immature ticks, respectively (Table 3). Importantly, only 11 ticks were collected from group A dogs during the whole study period, being nine alive (eight adults and 1 immature) and two dead adults. All ticks collected were morphologically identified as *R. sanguineus* group. Similarly, a 100% efficacy against fleas was calculated for each of the four assessments in which flea infestations were detected (Table 4). The mean flea loads in groups A and B were statistically different (p < 0.01) only at the end of the season (September and November 2011). All fleas collected were morphologically identified as *C. felis*.

**Table 3:** **Mean counts of and efficacy against adult and immature stages of *R. sanguineus *group ticks during the treatment period**

**Tick stage**	**Groups**	**Mean**	**2011**									**2012**			
			**Day 0**	**May**	*Jun 1	*Jun 2	**Jul**	**Aug**	**Sep**	**Oct**	**Nov**	**Jan**	**Feb**	**Mar**	**Apr**
Adult	A	Arithmetic	37.5	0.2	0.2	0.1	0	0.1	0	0	0	0	0	0	0
		Geometric	19.3	0.1	0.1	0.1	0	0.1	0	0	0	0	0	0	0
	B	Arithmetic	56.9	49.9	51.3	3.1	2.8	4.7	1.7	0.2	0.3	0.1	0.2	1.3	13.1
		Geometric	22.8	19.8	39.8	2.0	1.1	3.0	1.1	0.1	0.2	0.1	0.1	0.8	10.0
	Efficacy Ar. mean (%)		**-**	99.6	99.6	96.7	100	97.9	100	100	100	100	100	100	100
	Efficacy Ge. mean (%)		**-**	99.5	99.8	95.0	100	96.7	100	100	100	100	100	100	100
Immature	A	Arithmetic	0.1	0	0	0	0	0	0	0	0	0	0	0	0
		Geometric	0.1	0	0	0	0	0	0	0	0	0	0	0	0
	B	Arithmetic	0.1	1.8	4.3	106.3	0	0	0.3	0.1	0	0	0	0	4.5
		Geometric	0.1	0.8	2.8	25.7	0	0	0.2	0.1	0	0	0	0	2.6
	Efficacy Ar. mean (%)		**-**	100	100	100	n.a.	n.a.	100	100	n.a	n.a	n.a	n.a	100
	Efficacy Ge. mean (%)		**-**	100	100	100	n.a	n.a	100	100	n.a	n.a	n.a	n.a	100

*Sampling occurred in monthly intervals. As it was conducted at beginning and end of June, June appears twice.

Abbreviations: *n.a*. not applied, *Ar*. arithmetic, *Ge*. geometric.

**Table 4:** **Mean counts of *Ctenocephalides felis *and the efficacy during the treatment period**

**Group**	**Mean**	**2011**									**2012**			
		**Day 0**	**May**	*Jun 1	*Jun 2	**Jul**	**Aug**	**Sep**	**Oct**	**Nov**	**Jan**	**Feb**	**Mar**	**Apr**
A	Arithmetic	0.7	0	0	0	0	0	0	0	0	0	0	0	0
	Geometric	0.2	0	0	0	0	0	0	0	0	0	0	0	0
B	Arithmetic	1.3	0	0	0	0	0.1	0.2	0	0.4	0	0	0	0.2
	Geometric	0.5	0	0	0	0	0.1	0.1	0	0.2	0	0	0	0.1
Efficacy Ar. mean (%)		-	n.a.	n.a.	n.a.	n.a.	100	100	n.a.	100	n.a.	n.a.	n.a.	100
Efficacy Ge. mean (%)		-	n.a.	n.a.	n.a.	n.a.	100	100	n.a	100	n.a	n.a.	n.a	100

*Sampling occurred in monthly intervals. As it was conducted at beginning and end of June, June appears twice.

Abbreviations: *n.a*. not applied, *Ar*. arithmetic, *Ge*. geometric.

## Discussion

This study investigated, for the first time under field conditions, the efficacy of a collar containing 10% imidacloprid and 4.5% flumethrin for the prevention of tick-borne pathogen infections in southern Europe, where a range of canine vector-borne diseases are endemic [2,8]. The pre-trial prevalence of tick-borne pathogen infections (e.g., *A. platys*, *H. canis*, *Babesia* spp., and *B. vinsonii* subsp. *berkhoffii* genotype III) in dogs, just prior to the beginning of the study, is in agreement with the results of previous investigations conducted in the same study site (see, for instance, Refs. [4,9]). Importantly, the role of sheltered dogs as reservoirs of certain pathogens for pet dogs and humans should be taken into account. For example, *E. canis* and *A. platys* are potential zoonotic pathogens [1,35]. These data highlight the need for the development of long-lasting and cost-effective strategies for the prevention and control of canine vector-borne diseases [8]. In particular, the use of collars impregnated with acaricidal/repellent compounds has recently provided promising results, indicating that long-lasting protection of dogs against the most common canine vector-borne diseases can be achieved in most instances [5,18]. Furthermore, collars may have advantages over other formulations (e.g., spot-on), including prolonged efficacy, steady concentration of the active compounds over its lifetime, and increased owner’s treatment compliance (reviewed by [11]).

The efficacy of the imidacloprid/flumethrin collar has been previously evaluated against ticks (i.e., *R. sanguineus*, *I. ricinus*, *I. scapularis*, *D. reticulatus* and *D. variabilis*), mites (i.e. *Sarcoptes scabiei*), and lice (*Trichodectes canis*) under controlled conditions [11]. The results of this trial complement the outcomes of the previous studies and further confirm the efficacy of this collar as a prophylactic tool against the transmission of tick-borne pathogens. Indeed, the high overall efficacy (99.7%) against both adult and immature stages of ticks resulted in a high level of protection against *B. vogeli* and *A. platys*. The protection of dogs against ticks and their transmitted pathogens is important, particularly because these pathogens have the potential to lead to severe disease, especially in cases of co-infections [7,10]. Based on the results of the present study, the collar conferred 100% protection against *B. vogeli*, 91.1% against *A. platys* and 43.4% against *H. canis*. The observed differences in levels of protection against distinct pathogens are likely related to a range of factors inherent to the biology, ecology and transmission dynamics of such pathogens [1,10]. For instance, the low efficacy of the collar against *H. canis* infection registered in the present study was expected considering that this protozoan is not transmitted by tick bites, but orally when a dog ingests an infected tick [10,36]. Probably, *H. canis* infections detected in dogs from collared and control groups were associated with an increase in the number of *H. canis-*infected ticks in the environment during summer months, as previously documented [1,37]. While the efficacy of the collar against tick infestations (>99%) impacts on the likelihood of a dog ingesting a *H. canis*-infected tick, our findings suggest that integrated control strategies focused on dogs and the environment may be warranted. For instance, the level of tick infestation in the environment might be particularly high in dog shelters; therefore, the application of insecticides in the environment may be necessary to control high tick burdens in this particular situation.

Molecular evidence of *A. platys* infection has been reported in a range of tick species [38-40], even if the role of ticks as vectors of this bacterium has yet to be confirmed [41]. Remarkably, the very small number of *A. platys* infections in dogs from group A demonstrates the efficacy of the imidacloprid/flumethrin collar against this pathogen and, indirectly, reinforces the hypothesis that *R. sanguineus* group ticks may transmit *A. platys* to dogs, especially considering the high incidence of infection during the post-treatment phase recorded herein.

Certainly, the efficacy of the collar against *R. sanguineus* group ticks may potentially help in preventing infection with pathogens other than *B. vogeli* and *A. platys*, such as *E. canis*, as demonstrated under laboratory conditions [19]. In fact, the overall efficacy against both adult and immature ticks recorded in this study is relevant, considering that most pathogens harboured by *R. sanguineus* group ticks are passed transstadially (e.g., *H. canis* and *E. canis*), while only a few are transmitted transovarially (e.g., *B. vogeli* and *R. conorii*) [42-44].

## Conclusions

The long-term protection conferred by the imidacloprid/flumethrin collar against fleas, ticks and tick-borne pathogens is in agreement with previous studies, in which the efficacy of the collar was ascertained for up to 8 months [11]. However, it should be noted that, upon removal of the collars, the prevalence of tick-borne infections in dogs from group A increased dramatically (e.g., *A. platys*, up to 67.4%). This finding is consistent with previous observations [10] and advocates the need for continued control of dogs against tick infestation, so as to prevent tick-borne pathogen transmission. Indeed, the availability of safe and effective acaricidal and/or repellent products will undoubtedly assist in the establishment of long-term control programs to prevent transmission of vector-borne diseases to dogs and may also help to reduce potential risks for human health.

## Competing interests

The authors declare that they have no competing interests.

## Authors’ contributions

DO and DS conceived the research. DO, FDT, DS, GC, CC, EB, AG, RR wrote the first draft, contributed with data analysis and interpretation, and revised the manuscript. DO, FDT, AG, RR, GD, AD, DD, RL, VT worked in the field on sample collection and animal examination SD, MSL, VT, BQ, VL performed diagnostic analyses. GC performed the statistical analyses. All authors read and approved the final version of the manuscript.
